# Monarchs and Medicine

**Published:** 1899-01

**Authors:** 


### MONARCHS AND MEDICINE.

His Majesty Menelek, Emperor of Abyssinia, who, it is reported, intends to present a paper on Vaccination at the forthcoming International Medical Congress in Paris, is said to take an enthusiastic interest in medical science, particularly in surgery. His favorite amusement, it is reported, is to be present at the operations in the hospital established at Adis-Alala by the Russian Mission. He watches the performance with a critical eye, and a skillful piece of knife-play or a dexterous feat of manipulation excites his warmest admiration. His pleasure rises to the highest pitch when he is given an arm to hold during an amputation, or is otherwise allowed to give active assistance. His delight on such occasions finds vent in rapturous exclamations of "Oyu gut! oyu gut!"—expressions which we give with all reserve, but which are said to mean "Admirable! admirable!" He is careful never to miss any rare or difficult operation, and insists on being kept posted in all that goes on in the hospital. This enthusiasm for the healing art on the part of the Negus recalls the fact that the Queen of Portugal has been credited with a like taste. Many years ago we gave currrency to a report that Her Majesty had, after going through the regular curriculum of study, taken the degree of Doctor of Medicine. This statement was the means of eliciting an official contradiction from the Lord High Chamberlain of Portugal. On the strength of this contradiction we did our best to put the matter right, but it is much easier to start a statement of this kind on its travels than to stop it. The inaccurate information which we were misled into publishing has been in circulation ever since. After going the round of the press in Europe it crossed the Atlantic, and had a long run in the United States. As it has not been seen for some time, we had begun to hope that its long career had finally ceased. But there does not seem to be in the world of journalism a bourne from which no traveler returns; and it is with a feeling akin to dismay that we read in a recent number of a leading medical journal published in New York that "Marie (*sic*), the young Queen of Portugal, who has been known for some time to be engaged in the serious study of medicine, is now reported to have received her formal degree of Doctor of Medicine." It is added that Her Majesty's first patient is her husband, whom she is treating for obesity, and that so far the new M.D. is satisfied with the results of her treatment. The last statement is what Artemus Ward would have called a "goak" coeval with the original legend, and therefore long past the fireshness of youth.—*The British Medical Journal.*

